# Evaluation of the new 'Confident Body Confident Child' prevention resource for parents of pre-school children: a randomised controlled trial

**DOI:** 10.1186/2050-2974-2-S1-O31

**Published:** 2014-11-24

**Authors:** Laura Hart, Stephanie Damiano, Fiona Sutherland, Susan Paxton

**Affiliations:** 1 La Trobe University, Melbourne, Australia

## 

Confident Body Confident Child (CBCC) is a new resource to assist parents in providing a positive body image and eating environment for 2-6 year-old children. The print materials, website, poster and information session were developed from research on child risk factors for body dissatisfaction and disordered eating. This study evaluated the CBCC resource using a four arm RCT; A) CBCC resource + face-to-face information session, B) CBCC resource only, C) Nutrition resource only and D) waitlist control. Parent participants completed online self-report measures of child eating and media viewing habits, parental feeding practices, parent disordered eating and body dissatisfaction, knowledge of positive parenting strategies and stigmatising attitudes to shape and weight. 340 participants completed baseline measures and were followed-up after receiving the resource, 6-months and 12months after. Focussing on the first round of results, repeated measures analyses comparing baseline to post-test revealed that receiving the CBCC resource was associated with significant reductions in parents' appearance-based stigma and instrumental feeing practices. Parents reported high engagement with the CBCC resource and enjoying face-to-face sessions. Conversely, the nutrition resource only was associated with increases in unhealthy parent feeding practices, such as covert control. Implications for future resource use are discussed.

This abstract was presented in the **Parental Roles in Prevention and Support** stream of the 2014 ANZAED Conference.

